# Natural selection of the D614G mutation in SARS-CoV-2 Omicron (B.1.1.529) variant and its subvariants

**DOI:** 10.1016/j.omtn.2023.01.013

**Published:** 2023-02-13

**Authors:** Chiranjib Chakraborty, Abinit Saha, Manojit Bhattacharya, Kuldeep Dhama, Govindasamy Agoramoorthy

**Affiliations:** 1 Department of Biotechnology, School of Life Science and Biotechnology, Adamas University, Kolkata, West Bengal 700126, India; 2 Department of Zoology, Fakir Mohan University, Vyasa Vihar, Balasore 756020, Odisha, India; 3 Division of Pathology, ICAR-Indian Veterinary Research Institute, Izatnagar, Bareilly 243122, Uttar Pradesh, India; 4 College of Pharmacy and Health Care, Tajen University, Yanpu, Pingtung 907, Taiwan

**Keywords:** natural selection, Omicron, subvariant, D614G mutation

The COVID-19 pandemic has been a threat to human health and the global economy for over 3 years.^1^^,^^2^ The causative RNA virus, severe acute respiratory syndrome coronavirus 2 (SARS-CoV-2), belongs to the betacoronavirus family and was preceded by SARS-CoV and middle east respiratory syndrome (MERS).^3^ RNA viruses are capable of rapid mutation, producing different variants. Natural selection is the key driving force for viral evolution, especially RNA viruses, and SARS-CoV-2 is no exception to this rule.^4^

SARS-CoV-2 enters the host cell through an interaction between its surface spike protein (S protein) and the angiotensin-converting enzyme 2 (ACE2) receptor.^5^^,^^6^ The S protein is a large trimeric protein comprising three non-covalently joined subunits. Any mutation in SARS-CoV-2 S protein is of interest given its direct involvement in viral infectivity and pathogenicity. For the past two decades, S protein and its mutations have been studied extensively in SARS-CoV and MERS for potential therapeutic development.^7^^,^^8^ Among these mutations is D614G, a non-synonymous mutation at the 614 position of S protein.

The high prevalence of D614G suggests that this mutation confers a selective advantage. Indeed, the D614G mutation induces a conformational change in S protein, which becomes more open, increasing its binding affinity and interaction with ACE2.^8^ This, in turn, increases the infectivity of SARS-CoV-2.^9^ A correlation with positive selection is also often associated with high-frequency mutations. Over time, it has become clear that D614G confers positive selection and has thus become widespread (Figure 1).^4^^,^^9^ Moreover, D614G is associated with higher infectivity and comparatively lower mortality, which drive positive selection and a persistent presence in the human population.^9^^,^^10^

**Figure 1:**
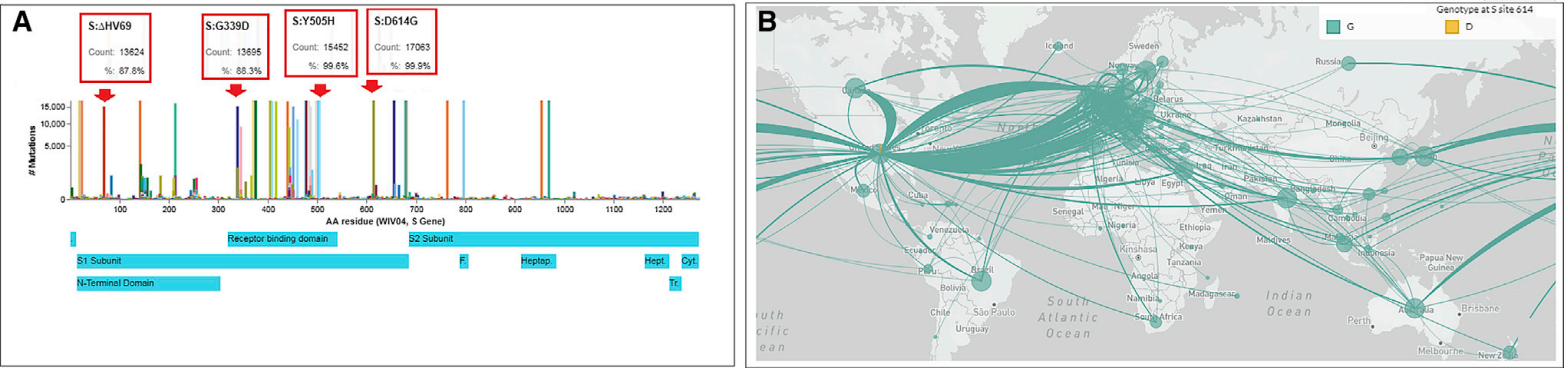
The frequency of D614G mutation in spike protein and its distribution worldwide (A) The D614G mutation shows one of the highest frequencies (99.9%) compared with other mutations in spike protein. (B) The distribution map of the D614G mutation shows the high occurrence of this mutation throughout the world. The figure was developed using the COVIDCG and GISAID databases.

The D614G mutation may also increase viral fitness. Yang et al. demonstrated that D614G helps SARS-CoV-2 to gain fitness by increasing folding stability.^11^ Majumdar and Niyogi also confirmed that mutations increase viral fitness, concluding that D614G is an essential mutation that enhances SARS-CoV-2 fitness.^12^ The D614G mutation also contributes to the increased transmission capacity of SARS-CoV-2. Leung et al. identified wild-type D614 clusters and mutant-type G614 clusters among sequences from ten countries.^13^ They estimated that the G614 mutant is 31% more transmissible than wild-type D614. Our previous *in silico* analysis supports this finding, as we found that SARS-CoV-2 variants with the D614G mutation are circulating worldwide.^4^

The SARS-CoV-2 Omicron (B.1.1.529) variant and its subvariants have become the dominant circulating variants worldwide, including circulating lineages B.1.1.529, BA.2, BA.2.75, BA.2.75.2, BA.2.12.1, BA.3, BA.4, and BA.5.^14^^,^^15^^,^^16^ Based on our findings, the D614G mutation is present at a high frequency in most Omicron subvariants (Figure 2).^4^ The prevalence of this mutation in Omicron subvariants can be attributed to natural selection, which plays a vital role viral evolution.^17^ Similarly, the Omicron (B.1.1.529) variant and its subvariants have been generated through natural selection during the evolution of SARS-CoV-2. Omicron subvariants have also acquired significant mutations through positive selection.

**Figure 2:**
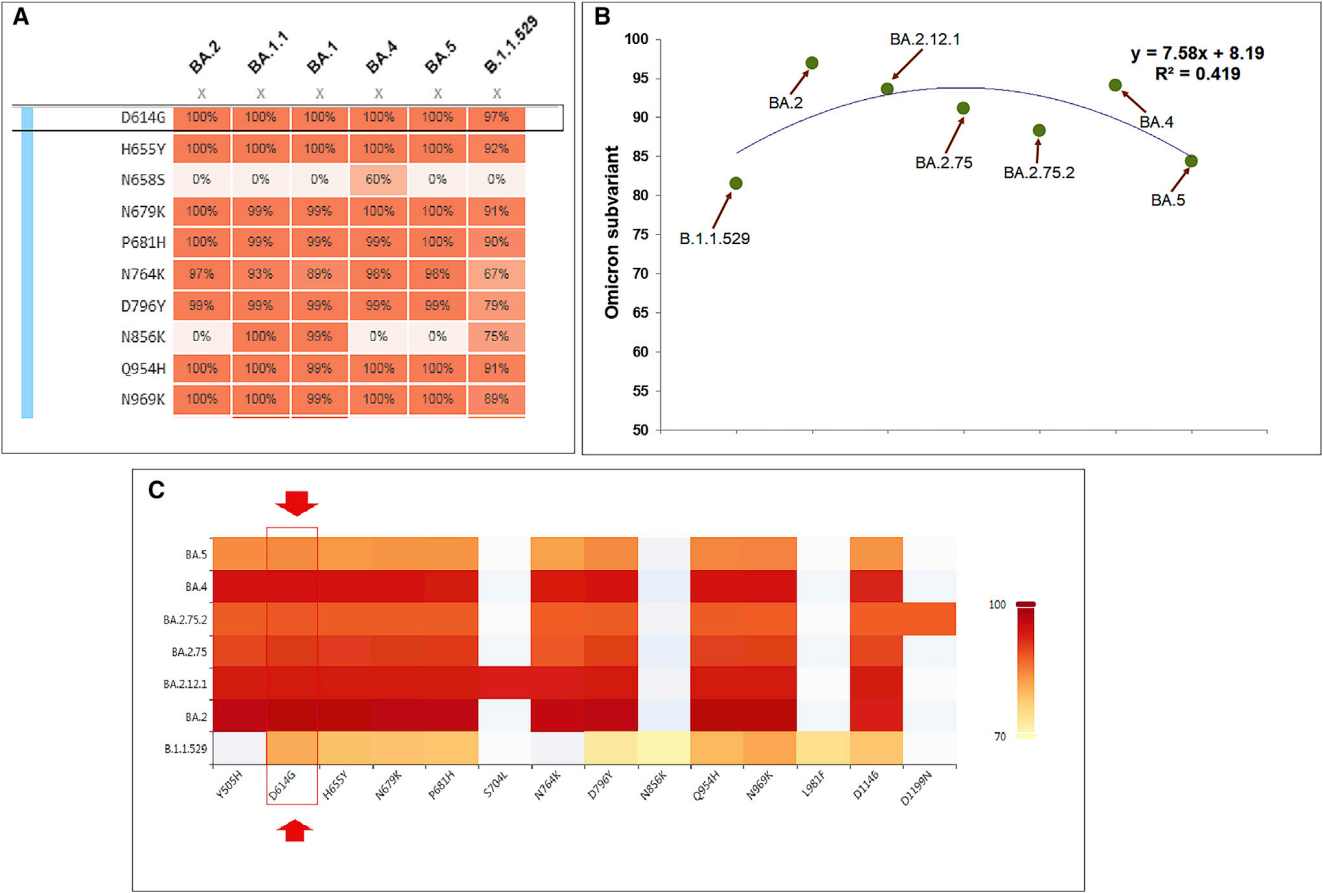
The occurrence of D614G mutation in the Omicron (B.1.1.529) variant and its subvariants (A) *In silico* analysis shows the D614G mutation present at a high frequency in the Omicron (B.1.1.529) variant and its subvariants B.1.1.529, BA.1, BA.1.1, BA.2, BA.4, and BA.5. (B) A statistical model represents the high frequency of D614G mutation in the Omicron (B.1.1.529) variant and its subvariants (B.1.1.529, BA.2, BA.2.75, BA.2.75.2, BA.2.12.1, BA.4, and BA.5). (C) A heatmap-like representation also shows the high frequency of the D614G mutation in the Omicron (B.1.1.529) variant and its subvariants. The figure was developed using the COVIDCG and GISAID databases and the National Genomics Data Center (NGDC)’s RCoV19 database from China.

The D614G mutation became dominant worldwide in a short period of time and precipitated many changes in the characteristic features of the S glycoprotein. The evolution of this particular strain has raised doubt among scientists regarding the efficacy of existing vaccines.^18^ Notably, the D614G mutation in Omicron and its subvariants might also be associated with the immune escape of SARS-CoV-2, increased transmission capacity, and re-infection, thus posing a substantial threat to humanity. Fortunately, despite the high transmission rate, the D614G mutation is not associated with increased mortality.

## Data availability statement

The authors confirm that the data supporting the findings of this study are available within the article.
